# Activity of isoflavone biochanin A in chronic experimental toxoplasmosis: impact on inflammation

**DOI:** 10.1007/s00436-022-07571-y

**Published:** 2022-06-17

**Authors:** Wafaa A. Aboukamar, Abeer A. Elhenawy, Manar S. Elmehankar, Manal A. Elzoheiry, Randa El-Gamal, Lamiaa M. Elabbasy, Heba Hany, Nairmen Nabih

**Affiliations:** 1 Department of Medical Parasitology, Faculty of Medicine, Mansoura University, 2 El Gomhouria Street, Mansoura, 35516 Egypt; 2 Department of Medical Biochemistry, Faculty of Medicine, Mansoura University, Mansoura, Egypt; 3 Department of Biochemistry, Faculty of Medicine, Almaarefa University, Riyadh, Saudi Arabia; 4 Department of Pathology, Faculty of Medicine, Mansoura University, Mansoura, Egypt

**Keywords:** *Toxoplasmagondii*, Me49 strain, Biochanin A, Isoflavone, Proinflammatory biomarkers, Inducible nitric oxide synthase

## Abstract

*Toxoplasma gondii* is a worldwide prevalent parasite. The infection has been linked to variable inflammatory effects including neuroinflammation. Biochanin A (BCA) is an isoflavone, known for its anti-inflammatory and anti-oxidative properties. In this study, we examined the effect of BCA on the brain and liver inflammatory lesions in a murine model with chronic toxoplasmosis. Mice were divided in to six groups: non-infected control, non-infected BCA-treated, and four infected groups with *Toxoplasma* *gondii* Me49-type II cystogenic strain: infected control, BCA (50 mg/kg/day)-treated, combined BCA/cotrimoxazole-treated and cotrimoxazole (370 mg/kg/day) alone-treated. Gene expression of tumor necrosis factor (TNF)-α, interleukin (IL)-1β, and inducible nitric oxide synthase (iNOS) was evaluated by quantitative real-time PCR in the brain and liver tissues. In the infected control group, an upregulation of TNF-α and IL-1β mRNA expression levels was found. However, a downregulation of iNOS expression was detected in the brain of infected control mice. In both BCA- and combined-treated groups, the brain and liver tissues showed significantly reduced inflammatory lesions compared to the infected control mice with inhibited TNF-α and IL-1β mRNA levels. The iNOS expression levels in the brain tissues of BCA group were significantly higher than the levels of the infected control group. BCA alone or combined significantly reduced *T. gondii* cyst count in the brain tissues. In conclusion, the anti-inflammatory activity of BCA was demonstrated in the brain tissues of mice with chronic toxoplasmosis with decreased TNF-α and IL-1β expression levels and increased iNOS expression levels.

## Introduction

*Toxoplasma gondii* (*T.* *gondii*) is an opportunistic parasite, causing little or no apparent disease manifestations in immunocompetent individuals. However, during congenital infection or immunodeficiency, toxoplasmosis could lead to serious outcomes (Denkers 1999). The worldwide prevalence of toxoplasmosis was estimated to range between 10 and 80% (Pappas et al. 2009).

The intracellular parasites such as *T.* *gondii*,* Leishmania* sp. and *Trypanosoma cruzi* were recognized to induce activation of the host cells’ mitogen-activated protein kinase (MAPK) signaling pathways and induction of proinflammatory cytokines in macrophages (Valère et al. 2003; Kim et al. 2005; Nogueira et al. 2015). MAPKs are a family of serine/threonine kinases that include stress-activated protein kinase/JNK, ERKs, and p38, which are essential regulators of immunity and associated with numerous cell functions such as cytokine expression, cell proliferation, and apoptosis (Lu et al. 2017).

Th1 immune response against *T.* *gondii* infection is mediated by proinflammatory cytokines including interferon-gamma (IFN-γ), tumor necrosis factor-alpha (TNF-α), and interleukin-1beta (IL-1β) (Suzuki and Remington 1988), inducing the infiltration of immune cells as CD8 + T cells into the brain (Schlüter et al. 2001). Inducible nitric oxide synthase (iNOS) with reactive nitrogen intermediates production has demonstrated a pivotal role in controlling chronic toxoplasmosis (Scharton-Kersten et al. 1997; Silva et al. 2009).

The expression of inflammatory cytokines is induced to keep the dormancy of *T.* *gondii*, although they could cause a subsequent neuromodulation and host behavior changes (Dunn 2006; Liesenfeld et al. 2011). Furthermore, inflammatory responses could affect uninfected neurons and disrupt the synaptic transmission (McCusker and Kelley 2013). Upregulation of pro-inflammatory biomarkers was implicated in the neuropsychiatric disorders (Lang et al. 2018). According to Mahmoudvand et al. (2016), the mRNA levels of TNF-α, IL-1β, and IL-6 were significantly increased in *Toxoplasma*-infected mice compared to uninfected mice. Those cytokines were described as contributing factors for hyperalgesia and neuroinflammation in mice brain tissues.

Biochanin A (BCA), 5,7-dihydroxy-4′-methoxyisoflavone, the methylated precursor of genistein, is found mainly in alfalfa and red clover (Lam et al. 2004). The flavonoids’ anti-inflammatory effects had been documented (Kole et al. 2011; Saviranta et al. 2011).

BCA inhibits the activation of MAPK pathway, leading to nuclear factor-κB (NF-κB)-driven inhibition of gene transcription and decreased expression of TNF-α, IL-1β, IL-6, iNOS, COX-2, and MMP-9 (Lam et al. 2004; Kole et al. 2011; Breikaa et al. 2013; Bhardwaj et al. 2014; Wu et al. 2018).

In this study, we investigated the effect of BCA, as a promising natural compound, on the inflammatory process of experimental chronic toxoplasmosis in the brain and the liver tissues. In addition, we aimed to assess the impact of BCA treatment on the parasitic load of the brain.

## Materials and methods

### Parasite

Mice were infected with *Toxoplasma* *gondii* Me49-type II cystogenic strain using a 22-gauge blunt feeding needle by intragastric inoculation of toxoplasmic cyst-containing brain homogenate, adjusted to 10 tissue cysts.

### Experimental design, animals, drugs, and dosing

Female Swiss Webster mice were purchased from the Medical Experimental Research Center, Mansoura University (age, 6 to 8 weeks; weight, 18 to 20 g), maintained at 20–23 °C in an air-conditioned laboratory and provided ad libitum with standard commercial pelleted diet and water. Mice were divided into six groups (10 mice each). Group (G) IA: uninfected control mice. G IB: uninfected BCA-control. G II: infected control, vehicle-treated (0.5 mL carboxy-methylcellulose). G III: infected treated with biochanin A (BCA), purchased from Sigma-Aldrich Co. (St. Louis, MO, USA). BCA (50 mg/kg body weight/day) was suspended in 0.5% carboxymethyl cellulose and administered by intragastric gavage (Moon et al. 2006; Breikaa et al. 2013). G IV: infected treated with combined BCA (50 mg/kg/day) and cotrimoxazole (GlaxoSmithKline, New Cairo, Cairo, Egypt), dissolved in DW in a total dose of 370 mg/kg, divided into two doses/12 h. G V: infected treated with cotrimoxazole alone as reference drug group (370 mg/kg divided into two doses/12 h). The drug was administrated at 8 weeks post-infection for 2 weeks. All mice were euthanized by pentobarbital sodium intraperitoneal injection (14 weeks post-infection). Brain hemispheres and liver were collected, washed in saline, and stored at − 20 °C for RNA extraction. This study was approved by local Institutional Review Board Ethical Committee, Faculty of Medicine, Mansoura University (code number: R/18.12.356).

### Parasitological examination

**Fig. 1:**
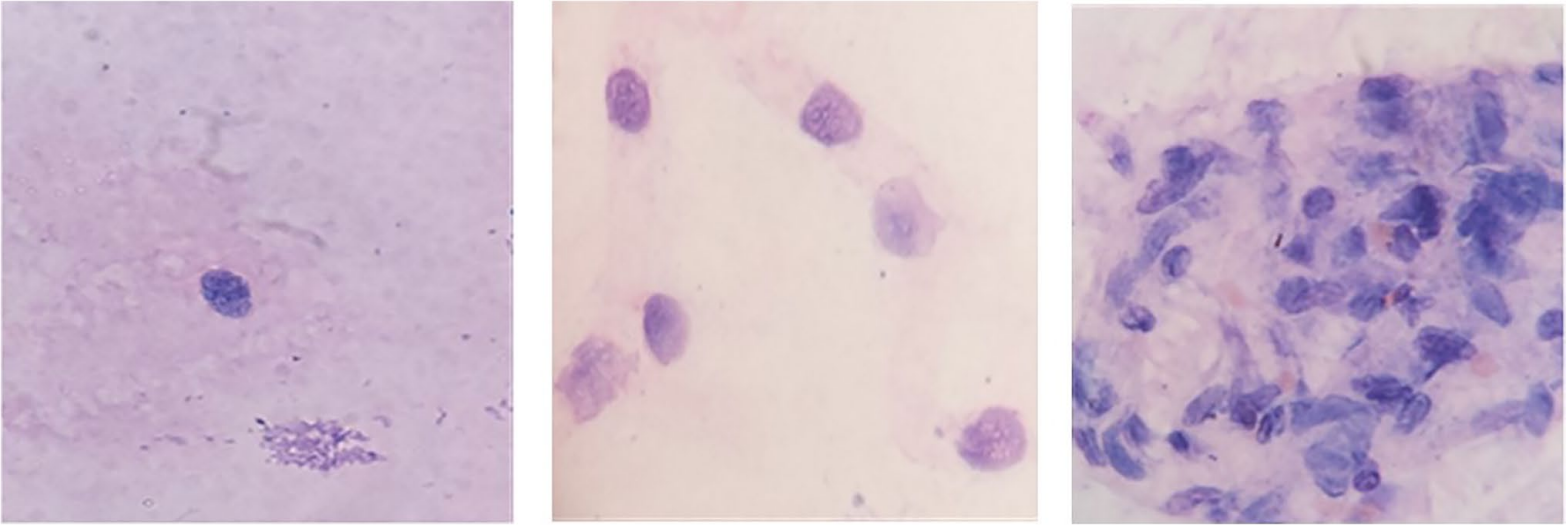
Brain tissue homogenates of infected mice stained with Giemsa stain showed variable aggregates of *Toxoplasma* cysts (× 1000)

The brain specimens from all mice groups were collected. Parts of each mice brain were homogenized in 1 mL phosphate-buffered saline pH 7.2. The brain cyst number was counted in 10 μL suspension per each homogenate (Kaňková et al. 2010). The cyst number was counted in 10/HPF and the mean number was calculated for each group. The size of the cysts was measured using an ocular micrometer (Araujo et al. 1996). Mice brain homogenates containing *Toxoplasma* cysts were mounted on microscopic slides, fixed, and stained by Giemsa stain (Garcia 2016) (Fig. 1).

### Histopathological examination

For histopathological assessment of the mice brain and liver specimens, parts of each organ was fixed in formaldehyde (10% in PBS) at pH 7.2, then processed by sectioning and hematoxylin and eosin (H & E) staining. A parasitologist and a pathologist (blinded) examined the slides. The following scoring system was used to estimate the severity of the histopathological lesions of the brain: 0: no lesion; 1: minimal lesion limited to localized perivascular cuffs with minor mononuclear cell infiltration; 2: mild lesion and local glial cell infiltration; 3: moderate lesion and focal necrosis; and 4: severe lesion, glial cell activation, and focally extensive necrosis (Tanaka et al. 2013). The scores for lesions of each area were assessed, and the total pathological score was used for data analysis. The scoring system used to estimate the severity of liver inflammatory changes was as follows: 0: no inflammatory activity; 1: minimal portal inflammation, lymphocytic infiltration, and spotty necrosis; 2: mild portal inflammation with lymphocytic infiltration and mild necrosis; 3: moderate portal inflammation, moderate lymphocytic infiltration, and noticeable hepatocellular change; and 4: severe portal inflammation, lymphocytic infiltration with possible fibrosis, and prominent diffuse hepatocellular necrosis (Batts and Ludwig 1995).

### Assessment of TNF-α, IL-1β, and iNOS gene expression by real-time PCR

Brain and liver tissue samples were homogenized by five strokes of liquid nitrogen. According to the manufacturer specifications, total cellular RNA was extracted using the QIAzol reagent (Qiagen, Hilden, Germany). The yield and purity of RNA were measured by NanoDrop 2000 (Thermo Scientific, MA, USA). Reverse transcription of 1 μg of RNA was done using SensiFAST™ cDNA Synthesis Kit (Bioline, London, UK). Quantitative real-time PCR (RT-qPCR) was carried out with HERA SYBR green PCR Master Mix (Willowfort, Birmingham, UK) in a total volume of 20 μL using Pikoreal 96 instrument (Thermo Scientific, MA, USA). The amplification reaction contained a 20 μL total volume mixture [10 μL of HERA SYBR green PCR Master Mix (Willowfort, Birmingham, UK), 2 μL of cDNA template, 2 μL (10 pmol/μL) gene primers, and 6 μL of nuclease-free water]. The reaction was 95 °C for 2 min, 40 cycles of 95 °C for 10 s, 60 °C for 30 s. The sequences of the used mouse primer pairs were as follows: TNF-α forward, 5′ TGAACTTCGGGGTGATCGGT 3′, reverse, 5′ GGTGGTTTGTGAGTGTGAGGG 3′ (Ref Seq: NM_001278601.1) and the product length was 99 bp; IL-1β forward, 5′ GCAACTGTTCCTGAACTCAACT 3′, reverse, 5′ GGGTCCGTCAACTTCAAAGA 3′ (Ref Seq: NM_008361.4) and the product length was 81 bp; iNOS forward, 5′ CAGCTGGGCTGTACAAACCTT 3′, reverse, 5′ CATTGGAAGTGAAGCGTTTCG 3′ (Ref Seq: NM_001313921.1) and the product length was 95 bp; and glyceraldehyde-3-phosphate dehydrogenase (GAPDH) forward, 5′ AGGTCGGTGTGAACGGATTTG 3′, reverse, 5′ TGTAGACCATGTAGTTGAGGTCA 3′ (Ref Seq: NM_001289726.1) and the product length was 123 bp; GAPDH was used as the control gene (Panina et al. 2018). The primer sets were designated using Primer 3 software (v.4.1.0) [http://primer3.ut.ee], and primer specificity was determined using the Primer-BLAST program (NCBI/primer-BLAST) [https://www.ncbi.nlm.nih.gov/tools/primer-blast/]. Primer sets were ordered from Vivantis (Vivantis Technologies, Malaysia). The products were inspected visually on a 3% agarose gel with ethidium bromide staining and the data were presented as the means of 3 independent experiments. Furthermore, the data for relative fold change of gene expression were presented in terms of relative quantification (RQ) of target mRNA, normalized in respect to the housekeeping gene (GAPDH) and relative to the control sample (calibrator). The RQ of mRNA expression was calculated using the comparative threshold method (ΔΔCt) (Livak and Schmittgen 2001).

### Statistical analysis

Data were analyzed using IBM-SPSS software version 20 (IBM Corp., Armonk, NY, USA) and GraphPad Prism version 8 (GraphPad Software Inc., CA, USA). Quantitative data were tested using Shapiro–Wilk’s test and expressed as mean ± SD. One-way ANOVA was used to compare data between groups followed by post hoc multiple comparisons, Tukey test. Data were considered statistically significant at *P* value < 0.05.

## Results and discussion

### Parasitological assessment of *Toxoplasma* cyst in the brain

As shown in Table 1, treatment with BCA (50 mg/kg/day) for 2 weeks significantly reduced the mean of *T. gondii* cyst count in mice brain tissues (*P* < 0.001). Combined BCA and cotrimoxazole significantly reduced the brain cyst count (*P* < 0.05). Reduction rates of 71.5%, 89.6%, and 76% were recorded in G III, G IV, and G V, respectively. No complete eradication of the cysts was achieved in any group. Cysts from both treatment groups (G III and G IV) were few and significantly smaller in size (*P* < 0.001) with irregular outlines than infected control mice.

**Table 1:** The main of *Toxoplasma* *gondii* cyst count and size in brain samples from infected mice groups

Parameter	Mice group				Test of significance	
	Infected control II	BCA group III	Combined group IV	Cotrimoxazole group V		
Cyst count/HPF#	27.10 ± 3.03	7.70 ± 1.76*	2.80 ± 0.918*	6.50 ± 1.35*	*F* = 317.620	*P* < 0.001
Cyst size (μm)	32.20 ± 3.35	21.10 ± 2.72*	13.20 ± 1.75*	20.90 ± 2.02*	*F* = 94.460	*P* < 0.001

Data are presented as mean ± SD. One-way ANOVA test: * indicates significant difference from infected control group II at *P* < 0.001. #The reduction rates compared to infected control group II were 71.5%, 89.6%, and 76% in groups III, IV, and V, respectively

A potential antimicrobial activity of BCA was reported (Dastidar et al. 2004). BCA showed anti-parasitic activity against *Leishmania chagasi* and *Trypanosoma cruzi* (Sartorelli et al. 2009). In this study, a potential anti-parasitic effect of BCA was demonstrated by significant lower toxoplasmic cyst count and size in the brain tissues. This effect could be attributed to the level of iNOS in the brain. An additional explanation could be linked to the inhibitory effect of BCA on the MAPK pathway. *T.* *gondii* possessed MAPK activity: TgMAPK-1 and TgMAPK-2 functional homologs, with a suggested role of TgMAPK-1 in parasite proliferation and stage differentiation (Brumlik et al. 2004).

Several potential mechanisms could explain BCA effect on *Toxoplasma* cyst count in the brain tissues. BCA was recognized to target cell cycle regulatory proteins particularly cyclin-dependent kinases and cyclins resulting in arresting cancer cellular growth (Seo et al. 2011). Its effect as COX-2 inhibitor could inhibit *T.* *gondii* proliferation (Pereira et al. 2019; Yu et al. 2019). BCA effect as MAPK kinase inhibitor could inhibit *T.* *gondii* MAPK and reduce the host cell invasion (Robert-Gangneux et al. 2000; Kole et al. 2011).

BCA selectively inhibited phosphodiesterase-4 activity with decreased intracellular Ca2 + (Ko et al. 2004). Ca2 + signaling is essential for the cellular *T.* *gondii* parasitism and replication (Hortua Triana et al. 2018). Ca2 + uptake and storage are crucially needed for the intracellular parasites as *T.* *gondii*, particularly during cellular invasion and dissemination (Vella et al. 2021).

### Histopathological examination findings in the brain and liver

**Fig. 2:**
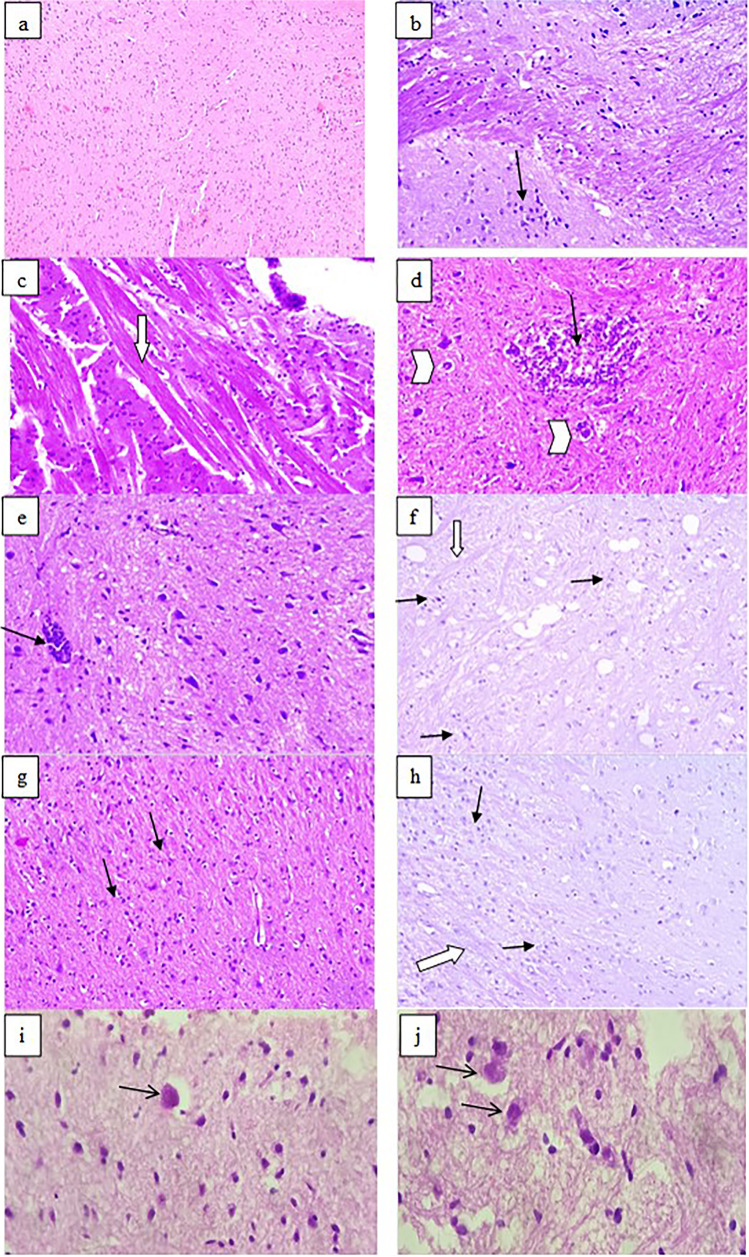
Effects of treatment regimens on the histopathological findings of brain specimens from all mice groups (H & E stained, × 200). **a** Normal brain tissues of uninfected mice. **b**–**d** Infected control; severe inflammatory infiltrate (black arrows), severe gliosis (white arrow), and giant cells (arrowheads). **e**, **f** Infected BCA-treated; mild to moderate inflammatory cellular infiltrates (black arrows) and mild gliosis (white arrow). **g**, **h** Infected combined-treated; **g** mild inflammatory cellular infiltrate (black arrows) and mild gliosis (white arrow). **i**, **j** Brain tissues of infected mice showed *Toxoplasma* cysts (H & E stained, × 400)

Brain sections from infected control G II showed marked inflammatory infiltrate. Severe gliosis was detected in most sections with lymphocyte infiltration and degenerated neural cells (Fig. 2b–d) compared to uninfected tissues. In both BCA- (G III) and combined-treated (G IV) mice, brain sections showed reduced inflammatory lesions compared to infected control mice, where inflammatory infiltrate ranged from mild to moderate degrees (Fig. 2e–h).

**Fig. 3:**
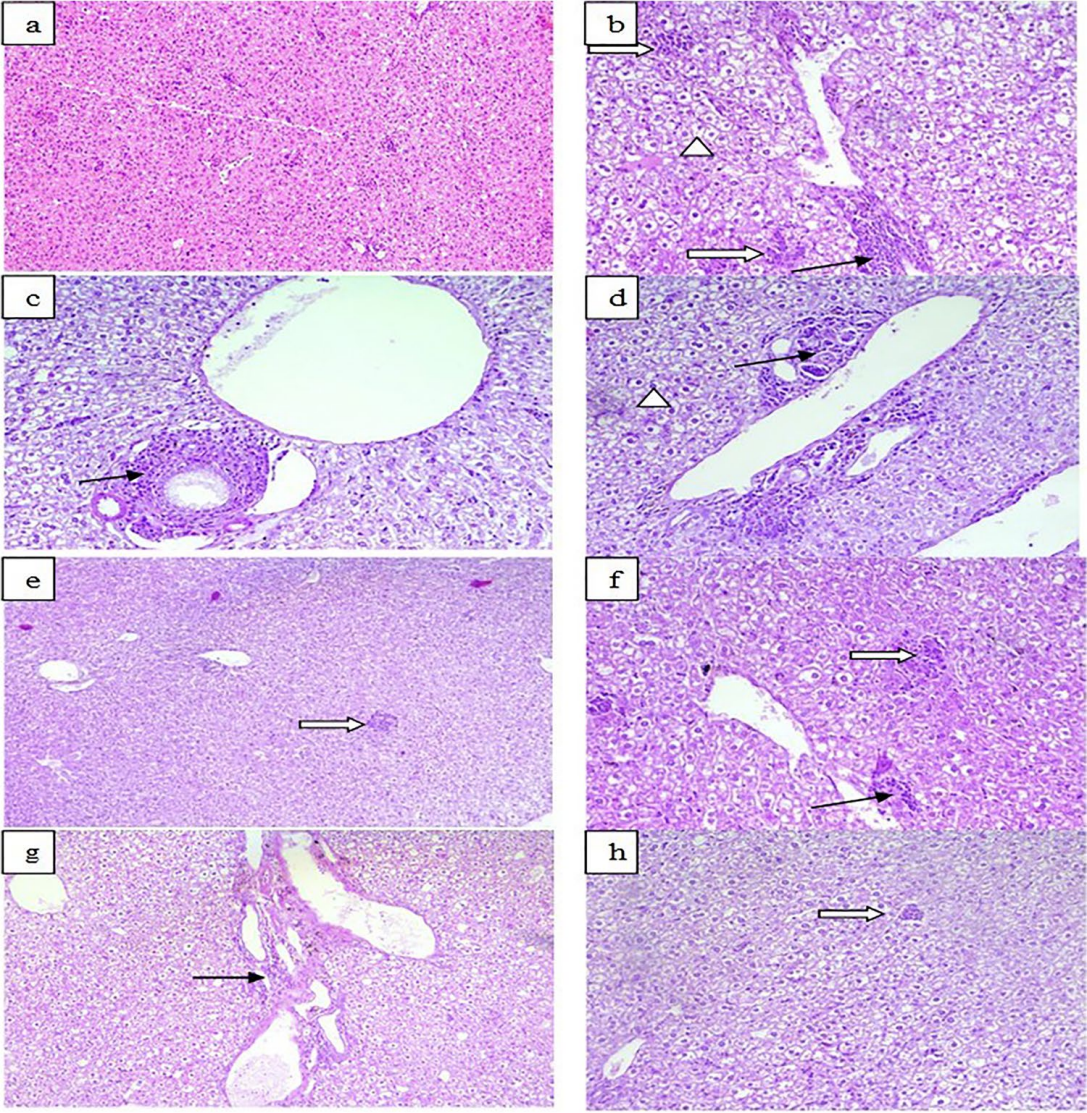
Effects of treatment regimens on the histopathological findings of liver specimens from all mice groups (H & E stained, **a**–**d**, **f**, **h** × 200; **e**, **g** × 100). **a** Normal liver tissue of uninfected mice. **b**–**d** Infected control, showed severe portal inflammation (black arrows), necroinflammatory injury (white arrow), and severe steatosis (arrowheads). **e**, **f** Infected BCA-treated; mild to moderate necroinflammatory injury (white arrows), mild portal inflammation (black arrow). **g**, **h** Infected combined-treated; mild portal inflammation (black arrow) and mild necroinflammatory injury (white arrow)

**Fig. 4:**
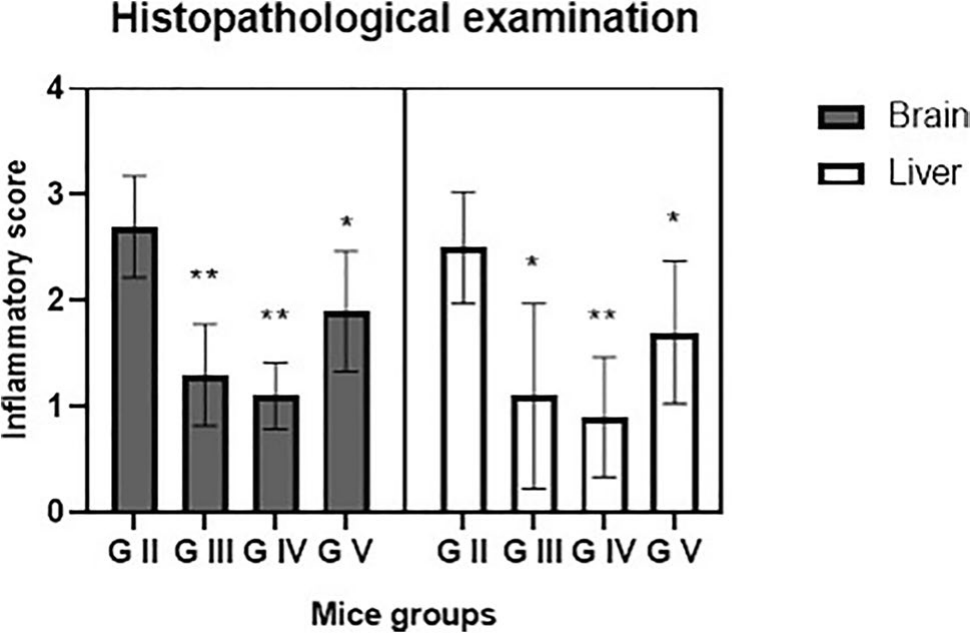
The mean of inflammatory score in the brain and liver tissues of mice with chronic toxoplasmosis. G II: infected untreated, G III: infected BCA-treated, G IV: infected combined-treated, G V: infected cotrimoxazole-treated. *P < 0.05 vs. G II. **P < 0.001 vs. G II

Liver tissues from infected mice showed moderate to severe inflammatory infiltrate with portal dilatation and periportal mononuclear infiltration of lymphocytes, histiocytes, and giant cells (Fig. 3b–d) compared to uninfected. In BCA- (G III) and combined-treated (G IV) mice, inflammatory infiltrate ranged from mild (60%) to moderate (40%) degrees (Fig. 3e–h). In combined-treated group, tissues of the brain and liver showed significantly reduced inflammatory scores (*P* < 0.001) compared to infected control (Fig. 4). In addition, in BCA group the inflammatory scores were significantly reduced (*P* < 0.05).

In this study, histopathological examination of the brain and liver tissues of BCA-treated and combined-treated mice showed milder inflammatory changes than the infected control group. Anti-inflammatory effects of BCA were demonstrated in previous studies (Kole et al. 2011; Saviranta et al. 2011; Wu et al. 2018).

### Effects of BCA on TNF-α, IL-1β, and iNOS gene expression in brain and liver tissues

**Fig. 5:**
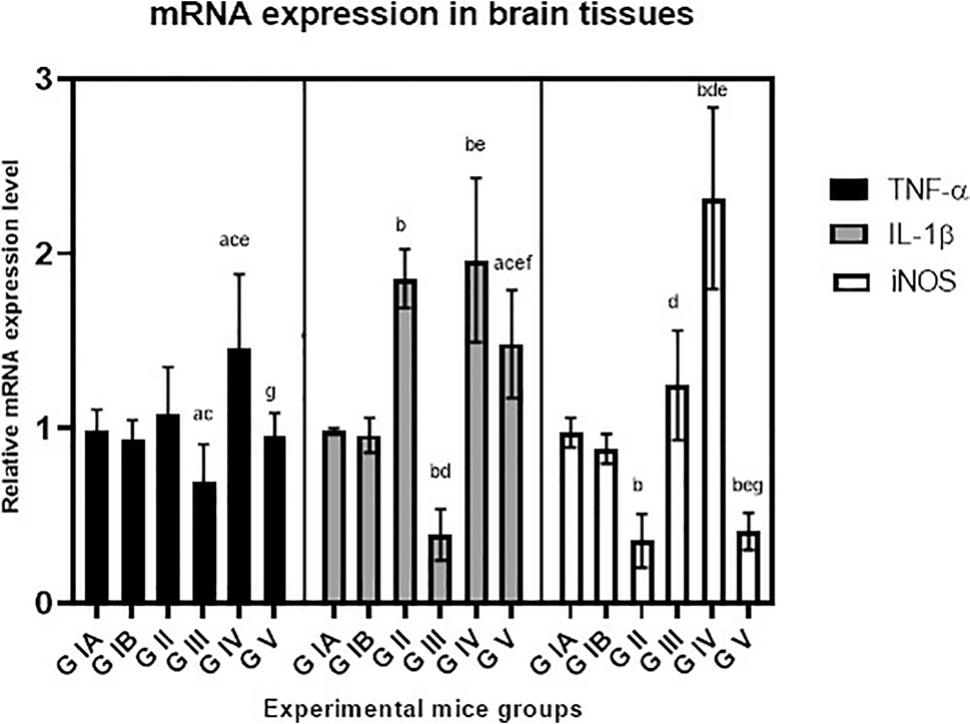
Effects of treatment regimens on TNF-α, IL-1β, and iNOS gene expression levels in the brain tissues of mice with chronic toxoplasmosis, analyzed by quantitative real-time PCR. G IA: uninfected control, G IB: uninfected BCA-treated. G II: infected control, G III: infected BCA-treated, G IV: infected treated with combined BCA and cotrimoxazole, G V: infected cotrimoxazole-treated. a: *P* < 0.05 vs. uninfected control group, b: *P* < 0.001 vs. uninfected group, c: *P* < 0.05 vs. infected control, d: *P* < 0.001 vs. infected control, e: *P* < 0.001 vs. BCA-treated, f: *P* < 0.05 vs. combined-treated, g: *P* < 0.001 vs. combined-treated

IL-1β expression levels were significantly increased in brain tissues of the infected mice compared to uninfected group (*P* < 0.001). Treatment with BCA (G III) significantly inhibited TNF-α and IL-1β expression levels (*P* < 0.05 and *P* < 0.001, respectively). However, in combined treatment G IV, no effect on TNF-α was detected compared to the infected control. The expression levels of iNOS gene were downregulated in the infected control group. Conversely, iNOS gene expression levels were significantly upregulated (*P* < 0.001) in BCA treatment group and in combined treatment group. The mRNA expression levels of all cytokines in the control mice treated with BCA were comparable to the untreated infected control. G V treated with cotrimoxazole showed no effect on TNF-α and iNOS mRNA levels compared to infected control G II; however, significantly reduced IL-1β mRNA levels were detected (*P* < 0.05) (Figs. 5 and 7a).

**Fig. 6:**
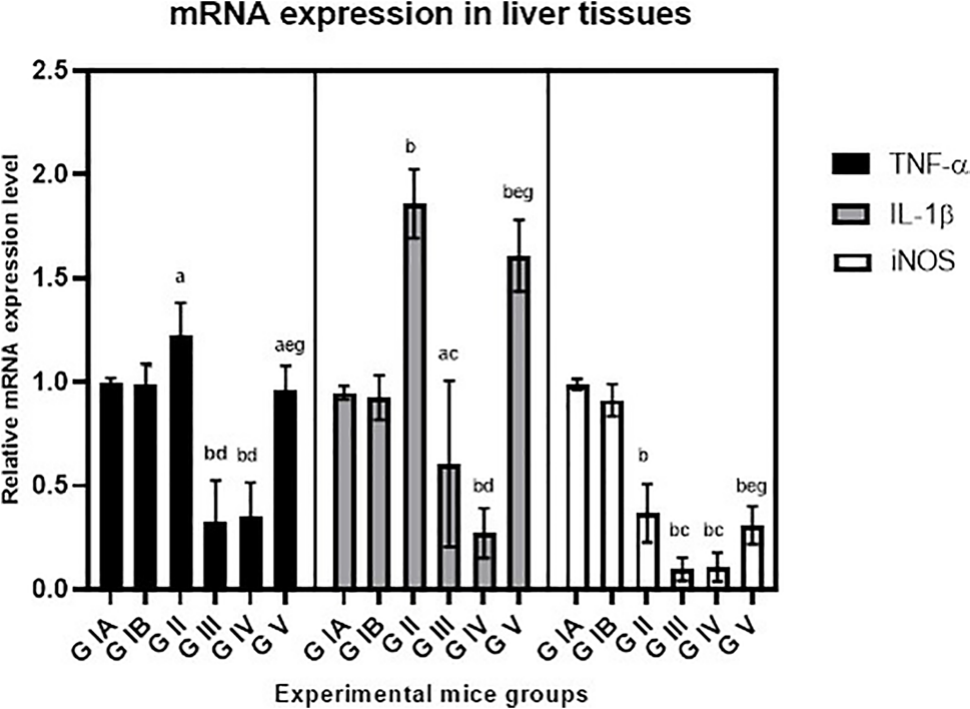
Effects of treatment regimens on TNF-α, IL-1β, and iNOS gene expression levels in liver tissues of mice with chronic toxoplasmosis, analyzed by quantitative real-time PCR. G IA: uninfected control, G IB: uninfected BCA-treated. G II: infected control, G III: infected BCA-treated, G IV: infected treated with combined BCA and cotrimoxazole, G V: infected cotrimoxazole-treated. a: *P* < 0.05 vs. uninfected control group, b: *P* < 0.001 vs. uninfected group, c: *P* < 0.05 vs. infected control, d: *P* < 0.001 vs. infected control, e: *P* < 0.001 vs. BCA-treated, f: *P* < 0.05 vs. combined-treated, g: *P* < 0.001 vs. combined-treated

**Fig. 7:**
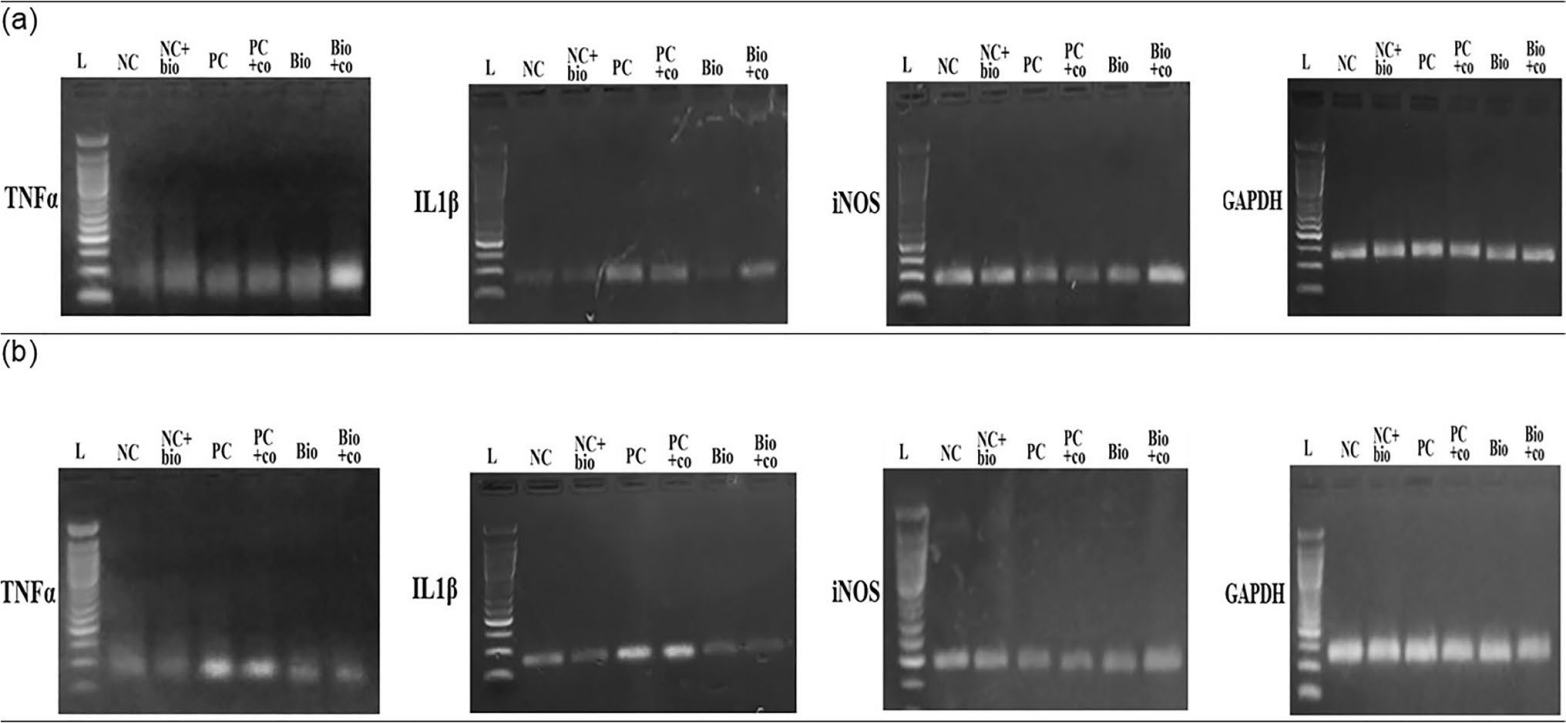
Gel electrophoresis of the real-time PCR products of the studied genes in different mice groups; **a** in the brain tissue samples, **b** in the liver tissue samples. Lane 1: 50 bp ladder (L), lane 2: negative control (NC), lane 3: negative control treated with biochanin A (NC + Bio), lane 4: positive control non-treated (PC), lane 5: positive control treated with cotrimoxazole (PC + co), lane 6: positive infected treated with biochanin A (Bio), and lane 7: infected treated with combined biochanin A and cotrimoxazole (Bio + co). IL1β qPCR product (81 bp), iNOS qPCR product (95 bp), TNFα qPCR product (99 bp), and GAPDH qPCR product (123 bp)

The expression levels of TNF-α and IL-1β were significantly increased in the liver tissues of infected mice (*P* < 0.001). Treatment with BCA alone or combined significantly decreased TNF-α and IL-1β mRNA levels in liver tissues (*P* < 0.001). BCA significantly inhibited the expression level of iNOS in both treatment groups (*P* < 0.001) (Figs. 6 and 7b). The mRNA expression levels of the cytokines in the control G 1B treated with BCA were comparable to the negative control G1A. G V treated with cotrimoxazole showed no effect on IL-1β and iNOS mRNA levels compared to infected control G II; however, significantly reduced TNF-α expression levels were detected (*P* < 0.05).

In this study, the assessment of proinflammatory biomarkers showed that BCA treatment reduced of TNF-α and IL-1β mRNA levels. Nevertheless, it upregulated the gene expression levels of iNOS in the brain tissues of mice with chronic toxoplasmosis. In agreement with our study, Wu et al. (2015) reported that BCA significantly decreased lipopolysaccharide (LPS)-induced mRNA expression of TNF-α and IL-1β.

IFN-γ is considered a major cytokine against *Toxoplasma* intracellular invasion and replication (Suzuki and Remington 1988). The role of iNOS (an IFN-γ-inducible protein) and the nitric oxide production has been established in the mice to keep the dormancy of *T.* *gondii* tissue cysts (Scharton-Kersten et al. 1997).

Meira et al. (2014) assessed IFN-γ and TNF-α activity in chronic toxoplasmosis patients versus reactivated cerebral and ocular toxoplasmosis patients. Patients from reactivated groups had low levels of IFN-γ when compared with those from chronic toxoplasmosis group although they had higher TNF-α levels. The most feared complication from chronic toxoplasmosis is the reactivation of latent infection; particular attention was given to TNF in our study. High TNF-α levels were implicated in the inflammation during reactivated toxoplasmosis suggesting the significance of BCA treatment of toxoplasmosis in decreasing TNF-α without affecting IFN-γ levels.

A study conducted on experimental bronchial asthma murine model showed that BCA suppressed the levels of cytokines, including TNF-α in the lung broncho-alveolar lavage fluid. However, it did not affect IFN-γ level (Ko et al. 2011).

Upregulation of proinflammatory cytokines expression, including iNOS, plays a major role in antibacterial response mechanisms. However, excessive production has been implicated in the impairment of the anti-oxidative system and exaggerated pathological inflammatory reactions (Matsumoto et al. 1998).

The iNOS expression levels were found to be downregulated in experimental parasitic infection with *Leishmania donovani*, an intracellular protozoan (Kole et al. 1999) and *Trichinella spiralis* nematode (Bian et al. 2001), which agreed with our findings. Our study showed that infection with *T.* *gondii* downregulated iNOS levels in infected control mice group. However, BCA treatment resulted in upregulation of iNOS gene expression levels in the brain with a significant decline in *Toxoplasma* cyst count.

According to Kole et al. (1999), doxorubicin-containing liposome combined with IFN-γ treatment of mice model infected *Leishmania donovani* resulted in upregulation of iNOS expression levels and subsequent reducing parasite numbers.

A high parasitic burden of *Toxoplasma* tissue cysts was found in the brain and might occupy up to 92% of the brain tissue (Berenreiterová et al. 2011). In addition, inflammatory infiltrates and foci of necrosis were observed independently of the *Toxoplasma* cyst sites.

The iNOS inhibitors could not reactivate chronic cerebral toxoplasmosis in BALB/c mice, but caused reactivation of chronic toxoplasmic encephalitis with a significantly increased intracerebral cyst count in *T.* *gondii* susceptible C57BL/6 mice (Schlüter et al. 1999). Low levels of iNOS expression were demonstrated in the tissues loaded with *Toxoplasma* cysts in mice infected with chronic toxoplasmosis, suggesting that histopathological lesions in organs were associated with parasite load rather than the level of iNOS (Schlüter et al. 1999). Previous findings agreed with our results. In our study, the histopathological scores in the brain were significantly lower in treated mice, with increased iNOS expression levels, than the non-treated mice.

In the present study, BCA treatment in chronic toxoplasmosis achieved a dual action: the suppression of TNF-α and IL-1β as proinflammatory cytokines, and a concomitant induction iNOS synthase. Increased iNOS expression levels in chronically infected mice with toxoplasmosis could be linked to the anti-parasitic activity of BCA. In another study, BCA inhibited both inflammatory and anti-inflammatory reactions in a *Salmonella*-infected mice model (Zhao et al. 2018).

Our data showed that BCA significantly inhibited TNF-α, IL-1β, and iNOS mRNA expression levels in the liver tissues. While *Toxoplasma* cysts are primarily present in the brain tissues during chronicity, parasitic load in the liver is far inferior to the brain burden at the eighth week post-infection (Autier et al. 2018). This could explain the controversial effects on iNOS expression, as BCA downregulates mRNA expression of iNOS in the liver tissues of mice with chronic toxoplasmosis. Anti-inflammatory effects of BCA were observed notably in liver tissues in this study. An interaction between hepatic stellate cells and *T.* *gondii* antigens was suggested to play a role in the liver pathology of toxoplasmosis-related hepatitis (Atmaca et al. 2013). Administration of BCA has been found to counteract hepatic damage caused by arsenic (Jalaludeen et al. 2016).

The combination of BCA and other drug therapies was assumed to be safe with a low possibility of alterations in the pharmacokinetics of the co-administered drugs (Arora et al. 2015). In our study, treatment with BCA alone downregulated TNF-α and IL-1β mRNA expression in the brain, while combined treatment resulted in higher levels. This could be attributed to cotrimoxazole effects that hindered back anti-inflammatory effects of BCA. Cotrimoxazole (trimethoprim/sulfamethoxazole) had a history of drug-drug interactions resulting in synergistic or antagonistic outcomes (Kaysadu et al. 2019). In this study, treatment with cotrimoxazole alone showed mild variable effects on the brain and liver tissue cytokines. According to Dubar et al. (1990), no significant effect was demonstrated by cotrimoxazole on IL-1 and TNF production, which agreed mostly with our findings.

In conclusion, the present study suggests potential anti-inflammatory effects of biochanin A in experimental chronic toxoplasmosis. These effects were demonstrated by attenuation of the pathological lesions in mice brain and liver tissues. Upregulation of iNOS expression levels in the mice brain tissues, associated with a significant decline in the parasitic cyst load, was also detected.
